# Heart rate biologging as a tool to understand the energetic needs of life history states

**DOI:** 10.1093/conphys/coag072

**Published:** 2026-09-28

**Authors:** Clare Stawski, Fritz Geiser, Gerhard Körtner, Shannon Currie

**Affiliations:** School of Science, Technology and Engineering, University of the Sunshine Coast, 90 Sippy Downs Drive, Sippy Downs, Queensland 4556, Australia; Centre for Behavioural and Physiological Ecology, Zoology, University of New England, Elm Avenue, Armidale, New South Wales 2351, Australia; Centre for Behavioural and Physiological Ecology, Zoology, University of New England, Elm Avenue, Armidale, New South Wales 2351, Australia; Centre for Behavioural and Physiological Ecology, Zoology, University of New England, Elm Avenue, Armidale, New South Wales 2351, Australia; School of BioSciences, University of Melbourne, Grattan Street, Parkville 3010, Australia

**Keywords:** Body temperature, daily energy expenditure, energetics, gestation, heart rate, lactation, metabolic rate, thermoregulation, torpor

## Abstract

Energy is vital for life, and the energetic needs of an organism can be quantified via its metabolic rate, for which measurements have traditionally been restricted to the laboratory. Alternatively, heart rate has been shown to be an excellent predictor of metabolic rate under laboratory conditions and can provide long-term measurements from free-ranging individuals at fine temporal resolution. We quantified the relationship between metabolic rate, heart rate and body temperature of a small arboreal marsupial, Krefft’s glider (*Petaurus notatus;* ~125 g) and how their energetic needs are affected by reproductive condition. Gliders were housed in a large outdoor aviary that experienced natural weather variations. We measured heart rate and body temperature continuously with internal implanted data loggers, and metabolic rates were intermittently measured in a laboratory with open flow respirometry. In addition to establishing a positive curvilinear relationship between heart rate and metabolic rate, these data were also used to calculate daily energy expenditure while gliders were in the outdoor aviaries. Daily energy expenditure decreased from winter to spring, indicating a strong seasonal influence, which likely resulted in lactating females utilizing less energy during spring than gestating females during winter. Understanding the energetic requirements of species under different life history states is vital for understanding their ecology and ultimately conservation management.

## Introduction

Energy is essential for animals to grow, survive, move and reproduce. Therefore, organisms are reliant on sources of energy, which can often shift between periods of abundance and deficit in response to both predictable seasonal weather changes and unpredictable environmental changes. These variations in resources often directly impact the physiological and behavioural state of an organism, which is vital to understand when modelling future impacts and making conservation decisions (Lavaud *et al.,* 2021). One way to quantify the changing energy requirements of an organism is by measuring their metabolic rate (MR). For resting animals heart rate (HR) has been shown to be an excellent predictor of MR under laboratory conditions and this relationship has been employed to extrapolate MR from measured HR of free-ranging individuals (Sapir *et al.,* 2010; O'Mara *et al.,* 2017). As many species of conservation concern are elusive and difficult to monitor, HR biologging can be employed to estimate the daily energy expenditure of individuals in their natural habitats, which can allow us to understand how animals respond to changes in their environment and also changes in individual state.

Animals living in habitats with distinct and predictable seasonal in weather and food availability typically show predictable long-term fluctuations in their MR, HR and body temperature (T_b_) (Toien *et al.,* 2011; Turbill *et al.,* 2011; Currie, 2018; Wascher *et al.,* 2018; Thiel *et al.,* 2019). In contrast, many animals that reside in unpredictable environments have evolved to be flexible in their physiology, exhibiting short-term fluctuations in MR, HR and T_b_ to compensate (Lubbe *et al.,* 2014; Levesque *et al.,* 2016; Hume *et al.,* 2020; Nowack *et al.,* 2020). This allows them to respond rapidly to harsh weather conditions and reduction in food and water resources. For example, a common response of mammals and birds to low food availability is to lower energy expenditure by entering a state of torpor. By reducing HR, MR and T_b_, torpor significantly decreases the energy needs of an animal (Ruf & Geiser, 2015).

In addition to the state of the environment, the state of an individual can also dictate their energy needs, for example body mass or reproductive state (Sørås *et al.,* 2022). Whereas heterothermy and torpor can afford animals energy savings in response to energetic bottlenecks and physiologically demanding periods (Ruf & Geiser, 2015), for many mammals fluctuations in T_b_ can be detrimental to reproduction (McAllan & Geiser, 2014). Therefore, some reproductive females restrict daily variations in T_b_ and MR, often with an overall increase in MR and HR during gestation and lactation to promote the growth and development of young (Levesque *et al.,* 2014a, 2014b, Stawski & Rojas, 2016, Thiel *et al.,* 2019, Wascher *et al.,* 2018). If changes in environmental temperature affect maternal temperature, this can significantly impact the development of young and also their behavioural and physiological traits as adults (Stawski & Geiser, 2020). Additionally, reproductive mammals generally have higher energy demands requiring them to source more food and thus increase their exposure to predators (McAllan & Geiser, 2014).

The aim of our project was to utilize heart rate biologging to understand the energetic needs of different life history states to highlight vulnerable stages for conservation management. We first aimed to quantify the relationship between MR, HR and T_b_ of a small arboreal marsupial, Krefft’s glider (*Petaurus notatus*), and subsequently how they respond to changes in reproductive condition while exposed to natural weather variations. Whereas sugar gliders (*Petaurus breviceps*) occur east of the Great Dividing Range along southeastern Australia, Krefft’s gliders occur along and west of the Great Dividing Range (Cremona *et al.,* 2021; Powley & Mikac, 2025). Therefore, many of the previous studies that we refer to have likely been conducted on Krefft’s gliders and not sugar gliders as stated in those studies (Cremona *et al.,* 2021). Both Krefft’s and sugar gliders are nocturnal and primarily feed on invertebrates and plant exudates (Smith, 1982). They are known to employ torpor opportunistically, particularly in response to cold temperatures, rain, unpredictable weather events and reduction in food availability (Körtner & Geiser, 2000; Christian & Geiser, 2007; Nowack *et al.,* 2015; Stawski & Simmonds, 2021). In the wild, pouch young are typically recorded during the Austral spring (September—November) with peak lactation from October–December, whereas in captivity, they have often been recorded breeding during the Austral winter (June—August; Suckling, 1984). Females have either twins or one young each breeding season. Most have only one litter per year, but two litters have also been recorded. Understanding how individuals manage their daily energy needs across life history stages will provide a significant understanding of the energy required for individual survival and offspring persistence.

## Materials and methods

### Animal care and logger programming/implantation

Eight captive Krefft’s gliders, three males (initial mass = 127.9 ± 10.7 g) and five females (initial mass = 122.6 ± 19.7 g), were measured for our study from June to November 2017. These gliders were originally captured in the wild in 2014 from two near locations, west of Dorrigo (30° 20′ 28′′S, 152° 42′ 50′′ E) and Imbota Nature Reserve (30° 34′ 24′′S, 151°43′ 15′′ E) in New South Wales, Australia (for details see Nowack & Geiser, 2016). While it is difficult to have complete certainty about gliders that were captured along the Great Dividing Range, for the purposes of this study, we will treat our gliders as Krefft’s gliders. The gliders were housed in two groups in outdoor enclosures (4.2 × 2.4 × 2.4 m) at the University of New England, Armidale, Australia. These outdoor enclosures each contained three wooden nest boxes, feeding platforms and branches for climbing. Under non-experimental conditions, the animals were fed a mixture of high protein baby cereal, high protein vitamin supplement (Wombaroo), egg, honey and water. Each group received 80 g of this mixture daily (approximate nutritional content: calories = 200–220 kcal, protein = 17–18 g, carbohydrates = 27 g, fat = 8 g) along with a variety of fresh fruit and water was provided *ad libitum*. All experimental procedures were approved by the University of New England Animal Ethics Committee (Authority number: AEC17–046) and New South Wales National Parks and Wildlife Service (Licence number: SL100791).

At the start of our study, all gliders were assumed to be non-reproductive as they had already been in captivity for three years with no prior reproductive events. However, one month after the surgeries, we discovered pouch young in one of the females and in the remaining females shortly after. Breeding in captivity during the Austral winter has been recorded in other studies (Suckling, 1984). We determined date of birth based on the first appearance of pouch young and their size and estimated reproductive state assuming an average gestational period of 16 days, an average period of lactation on the teat of 40 days, and an average period of lactation in the pouch of approximately 122 days (4 months) following (Smith, 1971). We used these dates to assess the measured variables against reproductive state. Figure 1 shows the timing of reproductive stages for each individual as well as days when metabolic rate measurements were taken.

**Figure 1:**
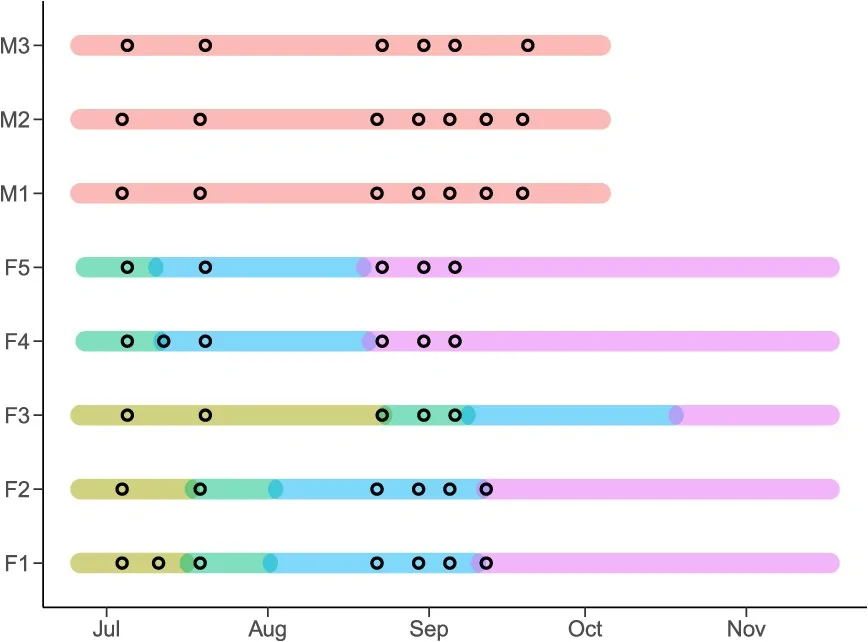
Timeline of the project. The timeline begins from the date of the initial surgery for implantation of biologging devices. Colours indicate the reproductive state of individuals (pink = males, yellow = non-reproductive females, green = gestating, blue = lactating on teat, purple = lactating) and open circles indicate dates on which metabolic rate measurements were conducted.

In these outdoor enclosures, the gliders experienced natural changes in the weather and photoperiod, although a section of each enclosure was roofed. As animals were captured from nearby field locations, they were accustomed to the local weather conditions in Armidale. This region experiences a cool temperate climate, with winter (June to August) temperatures ranging from 0 to 14°C and spring (September to November) temperatures ranging from 7 to 23°C. Both winter and spring are usually dry periods with little rain. We measured ambient temperature (T_a_) in the enclosures via the use of temperature data loggers (iButton thermochron DS1921G, Maxim Integrated Products, Inc., Sunnyvale, CA, USA; temperature resolution 0.5°C, accuracy 1.0°C), which were programmed to record every 10 minutes. To align the data with physiological measurements, we used linear interpolation between data points for T_a_.

Body temperatures and HR were initially programmed to be measured continuously during the entire experimental period. Core T_b_ was measured every two minutes via custom-made data loggers (D7 Dataloggers, T. Ruf, Vienna; temperature resolution 0.0625°C, accuracy 0.1°C) that were implanted intraperitoneally. To measure subcutaneous temperature (T_sub_) and HR, we implanted data loggers (HR-micro-HRT Star-Oddi, Gardabaer, Iceland; temperature resolution 0.032°C, accuracy 0.2°C) subcutaneously between the shoulder blades of the gliders. The loggers determined HR from measured ECGs over 3 seconds, but the original ECG was only saved intermittently for verification. Recording frequency of HR and T_sub_ measurements were programmed according to experimental needs and varied between every 5 minutes and every 30 minutes (see Metabolic rate measurements section for more detail).

Surgeries were performed under general oxygen/isoflurane anaesthesia administered via inhalation with a facemask (isoflurane in oxygen at 3–4% for induction and 0.5% for maintenance, flow rate 1–1.5 L min^−1^). The skin was disinfected prior and subsequent to the surgery using 70% ethanol and an iodine solution (Betadine). For the intraperitoneal surgeries, a small incision (~1 cm) was made to the skin and muscle layers, the logger (previously disinfected in ethanol) inserted, and the layers sutured together with resorbable suture (3.0 polyglycolic acid, Ethicon Inc., Johnson & Johnson Medical Pty Ltd, North Ryde, NSW, Australia). Following the recommendation by the manufacturer for inserting the HR loggers subcutaneously in small mammals, fur from between the shoulder blades was removed. A small incision (~1 cm) only to the skin layer was made with surgical scissors and the sterile logger inserted subcutaneously. The skin was also sutured with resorbable suture (3.0 polyglycolic acid, Ethicon Inc., Johnson & Johnson Medical Pty Ltd, North Ryde, NSW, Australia). Following suturing, a topical anaesthetic (10% Xylocaine, AstraZeneca Pty Ltd, North Ryde, NSW, Australia) was applied and the wound was further sealed with a spray-on bandage (Leuko Spray Bandage, BSN medical (Aust) Pty Ltd, Clayton, Vic, Australia). Recovery from anaesthesia was under 100% oxygen until the animal regained consciousness. The animal was then placed in a clean cage and monitored regularly over the following hours. Food and water were provided *ad libitum* and the animals were also provided with oral pain medication (0.1 ml of Meloxicam, 0.2 mg kg^−1^, Boehringer Ingelheim, North Ryde, Australia) and a once-off antibiotic injected subcutaneously (0.2 ml of Enrofloxacin, 2.5 mg ml^−1^, Baytril, Elanco, Indianapolis, Indiana, USA). The animals were given a week of recovery before any experimental procedures commenced and before the HR loggers began recording. The same surgical procedure was used to remove the loggers at the end of the experimental period to retrieve the recorded data.

Due to the natural curled resting position of the gliders stretching the dorsal skin, subcutaneously implanted HR loggers were rejected by five individuals after ~24 days. We subsequently implanted these HR loggers intraperitoneally in the three males using the protocol described above; we could not do the same for the females due to unexpected pouch young. This meant that we had inconsistent data for the males and females in this study, see Table 1 for a breakdown. As such, there was discontinuity between the T_b_ and HR measurements. We therefore only used T_b_ data from intraperitoneal loggers for analyses of daily T_b_ fluctuations and torpor use.

**Table 1:** Data available for analysis for each individual

**Glider**	**Heart rate and subcutaneous body temperature measurements**			**Internal body temperature measurements**		
	**Start/end dates**	**Duration (days)**	***N***	**Start/end dates**	**Duration (days)**	***N***
F1	04/07–13/09	71	6011	27/06–18/11	144	103 677
F2	N/A	N/A	N/A	27/06–18/11	144	103 678
F3	05/07–20/07	15	1404	27/06–18/11	144	103 677
F4	05/07–27/09	84	7007	28/06–18/11	143	102 959
F5	05/07–20/07	15	1409	27/06–18/11	143	102 958
M1	04/07–04/10	92	5066	27/06–4/10	99	71 280
M2	04/07–04/10	92	5068	27/06–4/10	99	71 280
M3	05/07–04/10	91	4846	27/06–4/10	99	71 280

(F = female, M = male).

### Metabolic rate measurements

To generate our model for predicting MR from HR, we needed to generate a dataset where HR and MR were measured simultaneously at a range of ambient temperatures. To do this, we measured oxygen consumption (V̇O_2_) as an indirect measure of MR using open-flow respirometry in all individuals, and across all reproductive states. For MR protocols 1 and 2 (see below) animals were placed into respirometry chambers in the afternoon (16:00–17:00) and removed the following afternoon (12:00–15:00). For the third MR protocol, animals were placed into the chambers at 07:00–08:00 and removed at 14:00–15:00. The three protocols that were used for metabolic measurements were as follows:

1. Low temperatures (T_a_ 0 to 10°C): Overnight the chambers were kept at 10°C and in the morning the T_a_ was decreased in 5°C steps to 0°C. Animals were held at each measurement temperature for 2–3 hours.
2. Mid-range temperatures (T_a_ 15 to 25°C): Overnight the chambers were kept at 15°C and in the morning the T_a_ was increased in 5°C steps to 25°C. Animals were held at each measurement temperature for 2–3 hours.
3. Thermoneutral zone (T_a_ 26.9 to 30.8°C) and high temperatures (T_a_ 32 to 38°C) measured during the austral winter: Animals were held at each measurement temperature from 26 to 30°C for 1 hour, whereas at T_a_ above 30°C animals were only held at each measurement temperature for 30 minutes to minimize heat stress.

Oxygen content of excurrent air was measured using a FOX analyser or an FC-1B analyser (both from Sable Systems International, Las Vegas, NV, USA, resolution 0.001%). The analysers switched between a reference channel and the animal channels every 3 min; two animal channels for the FC-1B analyser and three for the FOX analyser. Rotameters (Aarlborg 7908, New York, USA) equipped with needle valves were used to control the rate of airflow (~500 ml/min) into the chambers and airflow was measured using mass flow meters (Omega FMA-5606, Stamford, CT, USA). Air was dried before and after the animal chamber. Animals were kept in 1-L glass jars that were located inside a temperature-controlled cabinet. Temperatures within each of the individual chambers and the temperature-controlled cabinet were measured using calibrated thermocouples (±0.1°C, Omega DP116 digital thermocouple thermometer, Stamford, USA); these temperature data were also recorded to the computer via an A/D converter simultaneous to V̇O_2_ measurements. The data acquisition program was written by G. Körtner, using equation 3a of Withers to calculate V̇O_2_ (Withers, 1977). Sugar gliders were weighed before and after each experimental procedure to calculate a linear mass loss and mass-specific V̇O_2_ values. During respirometry measurements, HR was recorded every 5 minutes; outside of this time (when individuals were in their outdoor cages), HR was recorded every 30 minutes.

### Data analysis

#### Filtering of HR data

We recorded a total of 30 811 measurements of HR from seven gliders (four females, three males; one female had no heart rate data). Of these seven gliders, five had recording durations >70 days, with a maximum duration of recording of 92 days and a minimum duration of 15 days. HR recordings were verified from intermittent ECGs recorded by the loggers, which illustrated the variable HR predicted for small marsupials (Fig. 2). Heart rate was determined by averaging across as many unobscured consecutive heartbeats as possible within a 3-s recording window. StarOddi loggers tended to underestimate HR, this is potentially due to the technical specifications of the loggers and their method of dealing with variable HR within a measuring interval. StarOddi provide a built-in quality assurance method (QI = 0,1,2,3; QI3 being the lowest) based on a number of variables including HR variability and artefact. We assessed the quality of HR calculated via the logger versus via self-analysed ECGs within their assigned QI categories and found no clear pattern (Supplementary Table S1 in Supplementary Material 1); in fact the lowest QI category had a similar mean error compared to the two highest QI categories (QI3 = 49 ± 25 bpm versus QI0 = 32 ± 19 bpm and QI1 = 48 ± 25 bpm). Whereas data classified as QI3 had higher mean HR values, the distribution of QI3 data across measuring categories/reproductive states was generally consistent (Supplementary Fig. S1 in Supplementary Material 1). There was also no clear effect of logger placement on the quality of calculated HR when compared to ECG data; however, subcutaneously implanted loggers had a greater proportion of measurements classified as QI3 (Supplementary Fig. S2 in Supplementary Material 1). As the methods for quality classification were designed for laboratory rodents and apparently not strictly applicable to our data, we decided to include data from all QI categories in our final analysis, albeit after filtering based on predicted maximum HR and the minimum detectable HR from logger settings.

**Figure 2:**
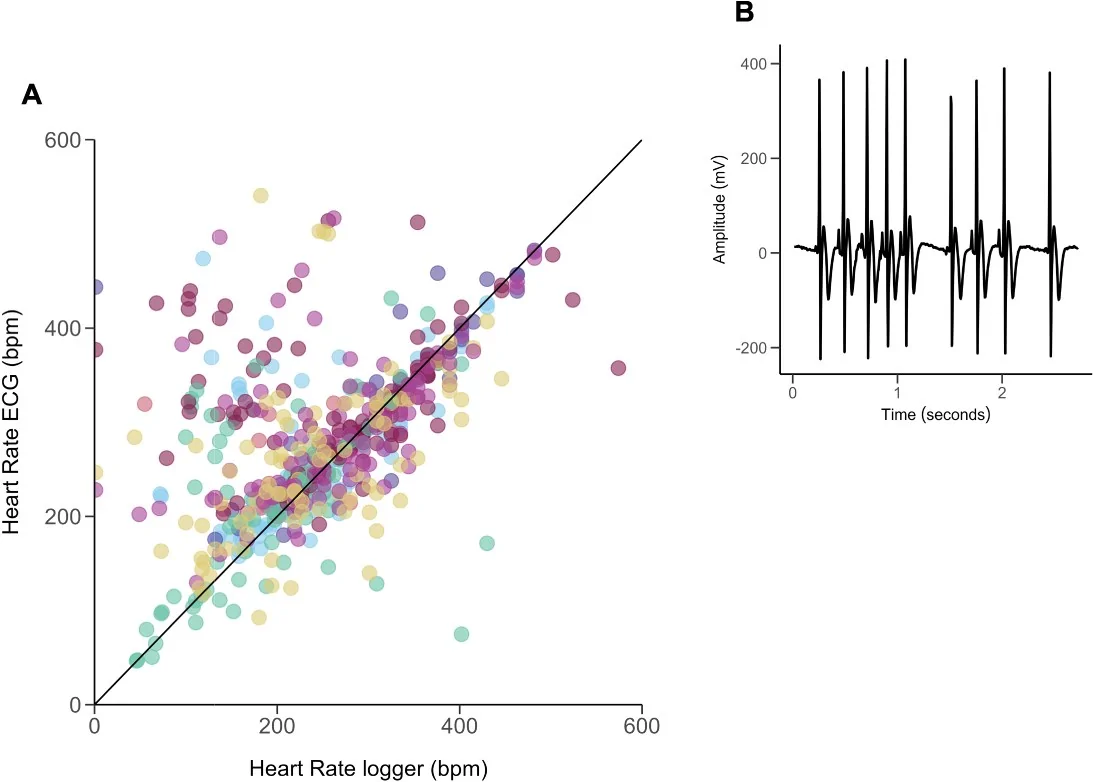
(**A**) Heart rate detected by the logger plotted against HR from verified ECGs. The solid black line indicates the line of equality. The logger tended to underestimate HR, and the effect was similar across individuals (colours represent different gliders). (**B**) Electrocardiogram of a sugar glider at rest showing the great deal of variability in instantaneous heart rate.

Initially, data were filtered between 20 and 655 bpm, as minimum HR detectable based on the logger settings was 20 bpm and maximal HR confirmed from ECG analysis was 653 bpm, which is higher than the maximum HR predicted from allometry (556 bpm) according to the equation max*f*_H_ = 375*B*^−0.19^ from Baudinette, (1978) using the mean body mass of our gliders prior to the start of the study (125 ± 16 g). This resulted in a minimum recorded HR of 21 bpm and maximum recorded HR of 653 bpm and reduced our dataset from 30 811 to 30 571 HR measurements.

#### Predictive modelling

To use HR as a proxy for MR, we first generated a generalized linear mixed effects model of HR against whole animal V̇O_2_. Due to the different timing of V̇O_2_ measurements and HR vs T_b_ loggers, time values were rounded to the nearest 5 minutes and matched accordingly. If two consecutive recordings of T_b_/VO_2_ were within 1 minute of the nearest 5 minutes, we calculated the mean of these (e.g. T_b_ measurements taken at 15:44 and 15:46 were averaged to correspond with HR measurement taken at 15:45). This resulted in a dataset of 3791 measurements from seven gliders, which was filtered to only include data where T_b_ was considered stable with <0.4°C between consecutive T_b_ measurements; excluding transitional periods between torpor and rest. To reduce autocorrelation, we then calculated the means of all variables for each combination of T_a_ and T_b_ (rounded to the nearest 1°C) per individual per day, thus reducing the final model dataset to 681 measurements. The mean number of measurements per individual was 97 (range 28–141). As torpor was only used by two animals during respirometry, we fitted a single model across all HR and V̇O_2_ data. We included individual as a random factor in the model. We accounted for the effect of T_b_ on the relationship between HR and V̇O_2_ by including an interaction term as there was a significant interaction between T_b_ and HR (*t*_675_ = 3.41, *P* < 0.001, Supplementary Fig. S3 in Supplementary Material 1), improving the predictive power compared to the model with no interaction term (conditional *R*^2^ = 0.52 vs 0.48). We then performed leave-one-group-out cross-validation (LGOCV) to determine the predictive power of our model.

The final model explained, on average, 52% of the variation in mean V̇O₂ across individuals (mean *R*^2^ = 0.52), with a root mean squared error of 57.9 ml O₂ h^−1^. To determine whether the quality classification of HR measurements impacted our model performance, we excluded all data with the lowest quality classification (QI3) and compared the model predictions to our model including all HR data. The exclusion of ‘low’ quality HR data had no impact on model predictions (Supplementary Fig. S4 in Supplementary Material 1). The full model was then used to predict MR of all individuals to compare measured and predicted MR using the function predict without including the random effect of individual (i.e. by setting re.form = *NA*). The final model only considered data for individuals resting within a respirometry chamber and therefore we cannot be sure of the relationship between HR and V̇O_2_ during activity. However, the range for both T_b_ and HR recorded during the activity phase at night and within the metabolic chamber during the rest phase were similar. Our model tended to underestimate V̇O_2_, with a mean difference between observed and predicted values of −11.45 ml O_2_ h^−1^ (range; −432.49 to 306.66 ml O_2_ h^−1^) (Fig. 3). Only two gliders entered torpor (once each) during respirometry measurements; however, the model still performed well when predicting V̇O_2_ during torpor. Predicted values across the complete torpor bouts, including transitional phases of entry and arousal, were only 26.5% lower on average than direct measurements of V̇O_2_.

**Figure 3:**
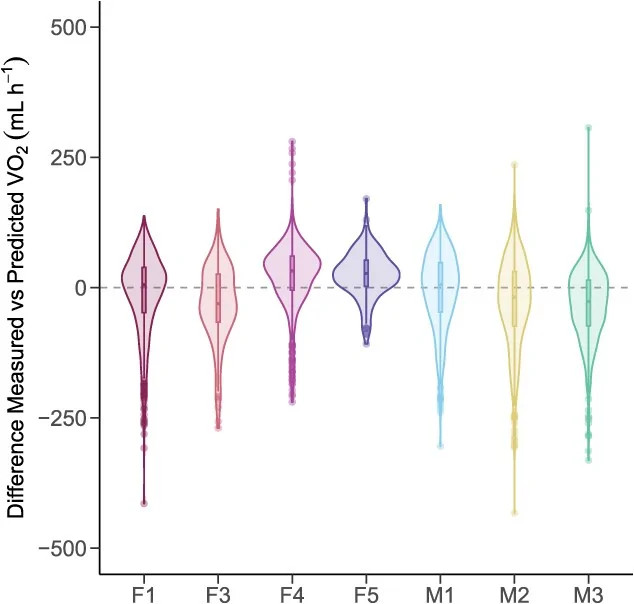
Distribution and range of calculated differences between measured oxygen consumption ($ \dot{\textsf{V}}\textsf{O} $_2_) and predicted $ \dot{\textsf{V}}\textsf{O} $_2_ for individual gliders. Violin plots show distribution of the data, box plots show median and interquartile ranges, whiskers extend to the range and outliers are represented by circles. Our model tended to underestimate $ \dot{\textsf{V}}\textsf{O} $_2_ indicated by median values below zero (dashed line). However, most data are distributed around the line of equality indicating good predictive power of the model. Model predictions were made using a general model, which excluded the random effects.

#### Daily energy expenditure

We estimated daily energy expenditure (DEE) of all animals in outdoor enclosures by summing the average V̇O_2_ predicted for each hour of the day over the entire 24 h period. On days when a full 24 h of recording was not available (i.e. when loggers were started after implantation, when they were explanted or when gliders were in the respirometry chambers), DEE was extrapolated to 24 h by dividing total V̇O_2_ by the number of hours recorded and multiplying this value by 24. To exclude periods of disturbance associated with handling/transfer, we removed data within 2 hours of animals entering or leaving the respirometry chamber. The resulting daily V̇O_2_ was then converted to MR (in kJ) assuming a mixed fuel source of 40% carbohydrates and 60% fatty acids (RQ = 0.76), using the conversion factor 20.1 (Withers, 1992).

#### Defining physiological states

To determine torpor bouts, we used T_b_ measurements from intraperitoneally implanted loggers and set a threshold of 30°C as used in previous studies on gliders (Körtner & Geiser, 2000; Nowack *et al.,* 2015). We acknowledge that the torpor cut-off method is arbitrary, yet have chosen this to be most comparable to previous studies and because gliders are too large to use previously defined torpor threshold equations (Willis, 2007). Heart rate was too variable to provide an adequate threshold for torpor; however, we generated a low HR threshold to characterize potential periods of low metabolism independent of T_b_. This threshold was set at 130 bpm—70% of estimated basal heart rate (BHR; 186 bpm). We estimated BHR using the allometric equation BHR = 106*B*^−0.27^ from Kinnear and Brown (1967) using the body mass (*B*) of our gliders (0.125 kg).

#### Statistical analyses

All raw data used for statistical analyses are in Supplementary Materials 2 and 3. Means are presented ± standard deviation, with ‘*n*’ representing the number of individuals and ‘*N*’ the number of observations. R Studio (2023.12.1) was used to conduct all data analyses (R version 4.3.2; The R Foundation for Statistical Computing, 2023). We used linear mixed effects models to determine the relationship between HR and MR (‘lmer’ package (Bates *et al.,* 2015) and performed bootstrapping to determine the 95% confidence intervals of our model predictions. We used linear mixed effects models with Satterthwaite’s method for estimating degrees of freedom (‘lmerTest’ package (Bates *et al.,* 2015, Kuznetsova *et al.,* 2017)) to determine which measured factors (reproductive state, T_a_, date, time of day, sex) affected the response variables (daily T_sub_ range, DEE, VO_2_ and HR). Time of day (day, night, dusk, dawn) was classified using nautical dawn, sunrise, sunset and nautical dusk as calculated from the package ‘suncalc’ (Thieurmel & Elmarhraoui, 2022) with ‘day’ consisting of anytime between sunrise and sunset and ‘night’ consisting of times between nautical dusk and nautical dawn. Each calendar date was adjusted to represent the 24 hours from dusk until dusk, as this is most biologically relevant for nocturnal species. Repeated measures on the same individual were accounted for by including individual as a random effect. Finally, to determine if there were differences between reproductive states and time of day for the measured variables, we ran pairwise comparisons between each group using Tukey–Kramer adjustment with Kenward Roger adjustment of degrees of freedom (package ‘emmeans’ (Lenth, 2025)).

## Results

Across our study, HR from active/resting gliders in outdoor aviaries ranged from a mean daily minimum of 70 ± 39 bpm up to mean daily maximum of 482 ± 72 bpm, with an overall mean daily HR of 252 ± 42 bpm. This corresponded to a mean estimated DEE of 89.7 ± 15.4 kJ d^−1^ (*n* = 7, *N* = 360) and an average daily T_b_ of 36.2 ± 0.6°C. As expected for nocturnal species, mean HR was significantly higher at night than during the day, and higher at dusk than any other time of the day (Tukey-adjusted *post hoc*, all *P* < 0.01) (Fig. 4A). The same pattern was clear for mean T_b_, with dusk revealing significantly higher and less variable T_b_ than any other time of the day (Tukey-adjusted *post hoc*, all *P* < 0.001) (Fig. 4B). Consequently, estimated DEE followed a similar pattern, although *post hoc* analysis revealed that each time of day was significantly different from every other. Heart rate and therefore estimated DEE were most variable at dawn and dusk, but T_b_ was most constrained at dusk.

**Figure 4:**
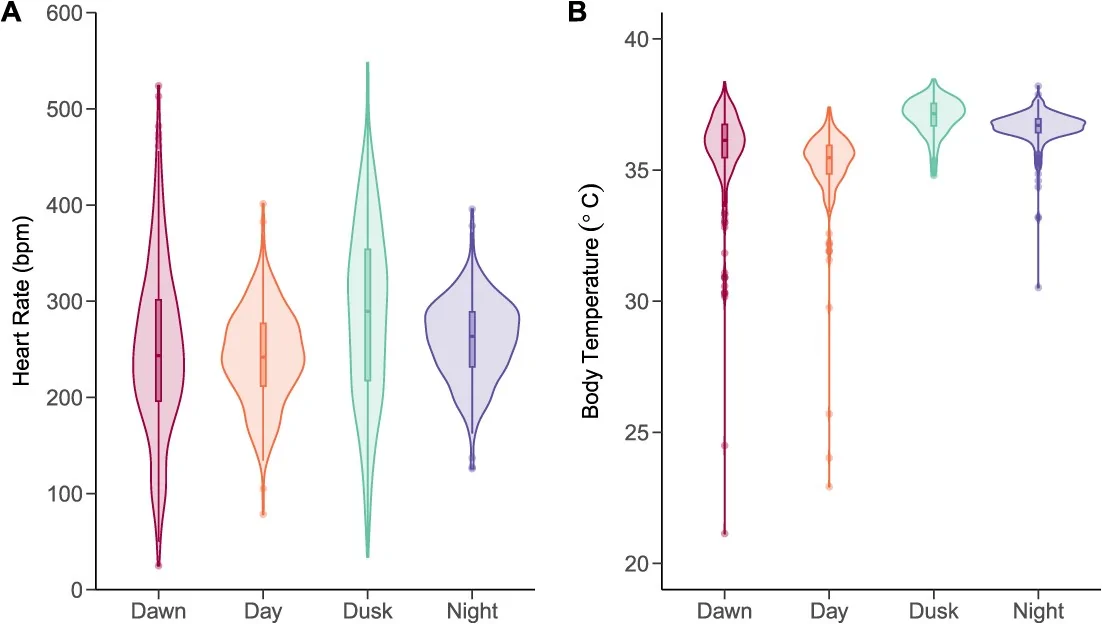
(**A**) Mean heart rate and (**B**) mean body temperature across all gliders as a function of time of day. Violin plots show the distribution, with median and interquartile ranges shown in the internal boxplots, outliers are represented by circles beyond the whiskers, which denote the data range. Heart rate and body temperature were significantly higher at dusk than any other time of the day.

We found that gestation was the most energetically costly period, with the highest estimated DEE (Fig. 5A) and least variation in daily T_b_ (Fig. 5B). Tukey-adjusted *post hoc* testing confirmed that lactating females (*P* < 0.01), those with young on the teat (*P* < 0.05), and males (*P <* 0.05) had significantly lower estimated DEE than gestating females, while all other pairwise comparisons were non-significant (*P* > 0.26). Higher estimated DEE in gestating females corresponded to significantly less variation in daily T_b_ (Tukey-adjusted *post hoc P* < 0.05) and the defence of a slightly higher daily mean T_b_ than lactating or non-reproductive females (Fig. 5). There were no significant differences found between non-reproductive females and both lactating groups (all Tukey-adjusted *post hoc P* > 0.506), and males were found to have substantially greater variation in daily T_b_ than females irrespective of reproduction (all Tukey-adjusted *post hoc P* < 0.01). The patterns observed for daily variation in T_b_ were not reflected in the daily variation in HR as we found no significant difference across all reproductive groups (Supplementary Table S2 in Supplementary Material 1).

**Figure 5:**
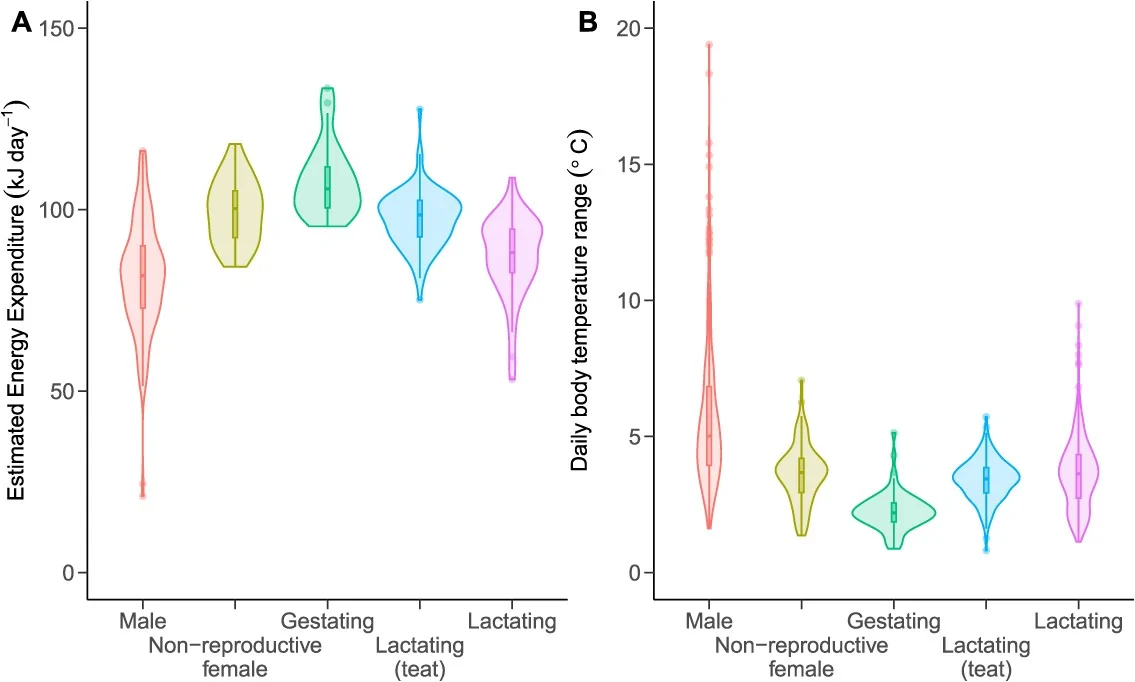
(**A**) Daily estimated energy expenditure and (**B**) daily body temperature range as a function of reproductive state. Violin plots show the distribution, median and interquartile range (box). Estimated daily energy expenditure was significantly higher in gestating females than males and females during both stages of lactation. Males exhibited significantly greater daily body temperature variation compared to all female reproductive states. Among females, gestating females had lower body temperature ranges than all other reproductive states.

From winter through to spring estimated DEE showed an overall decrease (Fig. 6), indicating the strong negative relationship between estimated DEE and mean daily T_a_ (β = −0.91 ± 0.16 SE, *t* = −5.82, *P* < 0.001). This pattern of decreasing estimated DEE with increasing T_a_ was also reflected in comparisons between reproductive state as there was a significant influence of mean daily T_a_ on differences in estimated DEE between males and gestating females (Tukey-adjusted *post hoc P* < 0.05), and gestating and lactating females (Tukey-adjusted *post hoc P* < 0.05). We also found that much of our data for lactation was within the period of highest daily mean T_a._

**Figure 6:**
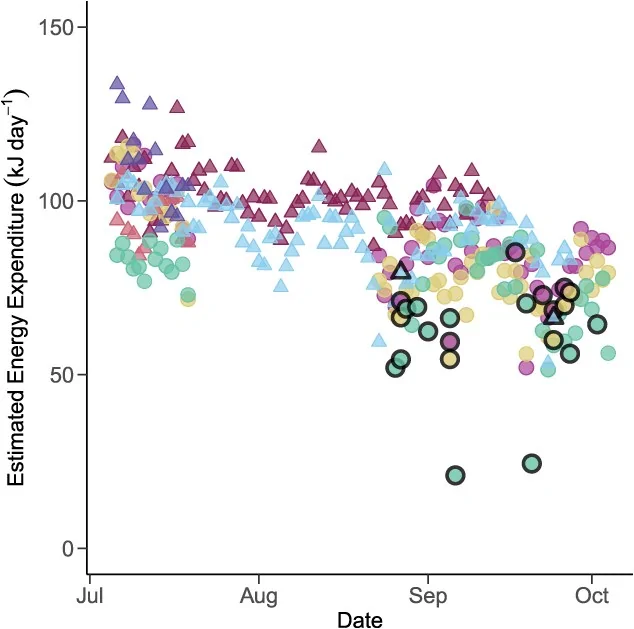
Estimated daily energy expenditure (kJ day^−1^) of individual gliders throughout the study period. Colours denote different individuals, data from females are represented by triangles and males are represented by circles. Days where torpor was used are highlighted by symbols with a black border.

Interestingly, although the degree of daily T_b_ variation differed significantly between the sexes, it was not related to daily minimum T_a_ for either sex (*P* = 0.41). Yet, daily fluctuations in T_b_ peaked between August and October (Fig. 7) corresponding with periods of low minimum T_a_, as would be expected for typical torpor use. Gliders used torpor on 23 days within their enclosures with a total of 39 torpor bouts recorded with all three males employing torpor (37 torpor bouts) and only one lactating female (two torpor bouts). During torpor, HR fell to an average minimum of 64 ± 31 bpm (*n* = 3, *N* = 20) for males. In the lactating female, HR fell to a minimum of 46 and 75 bpm for the two torpor bouts she entered. The absolute minimum HR during torpor was 23 bpm. The average HR across all complete torpor bouts (including partial entry and arousal) was 121 ± 30 bpm (*n* = 4, *N* = 22), which was 68% of the basal HR predicted from allometry (178 bpm). For males, the mean minimum torpor T_b_ was 26.2 ± 3.4°C (*n* = 3, *N* = 37; absolute minimum torpor T_b_ = 17.8°C). The minimum torpor T_b_ for the female for the two torpor bouts were 28.4 and 29.2°C. Estimated DEE was significantly lower on days when gliders entered torpor (β = −20.93 ± 2.42 SE, *t* = −8.66, *P* < 0.001) alongside a significant positive linear relationship between estimated DEE and daily minimum T_b_ (β = 3.65 ± 0.24 SE, *t* = 15.31, *P* < 0.001). Torpor use resulted in an overall energy saving of ~28.5 kJ per day on average; more than 30% less than mean estimated DEE on days without torpor (91.5 ± 13.6 kJ). The maximum estimated instantaneous energy savings from torpor was 31.3 kJ for a single torpor bout; 84.2% less that estimated resting energy expenditure over the same timeframe (mean instantaneous savings = 7.4 ± 6.9 kJ or 63.3 ± 14%). Maximum daily energy saving from torpor was up to 112.5 kJ per day when we compared minimum estimated DEE on torpor days to maximum estimated DEE when no torpor was used (21.0 vs 133.5 kJ).

**Figure 7:**
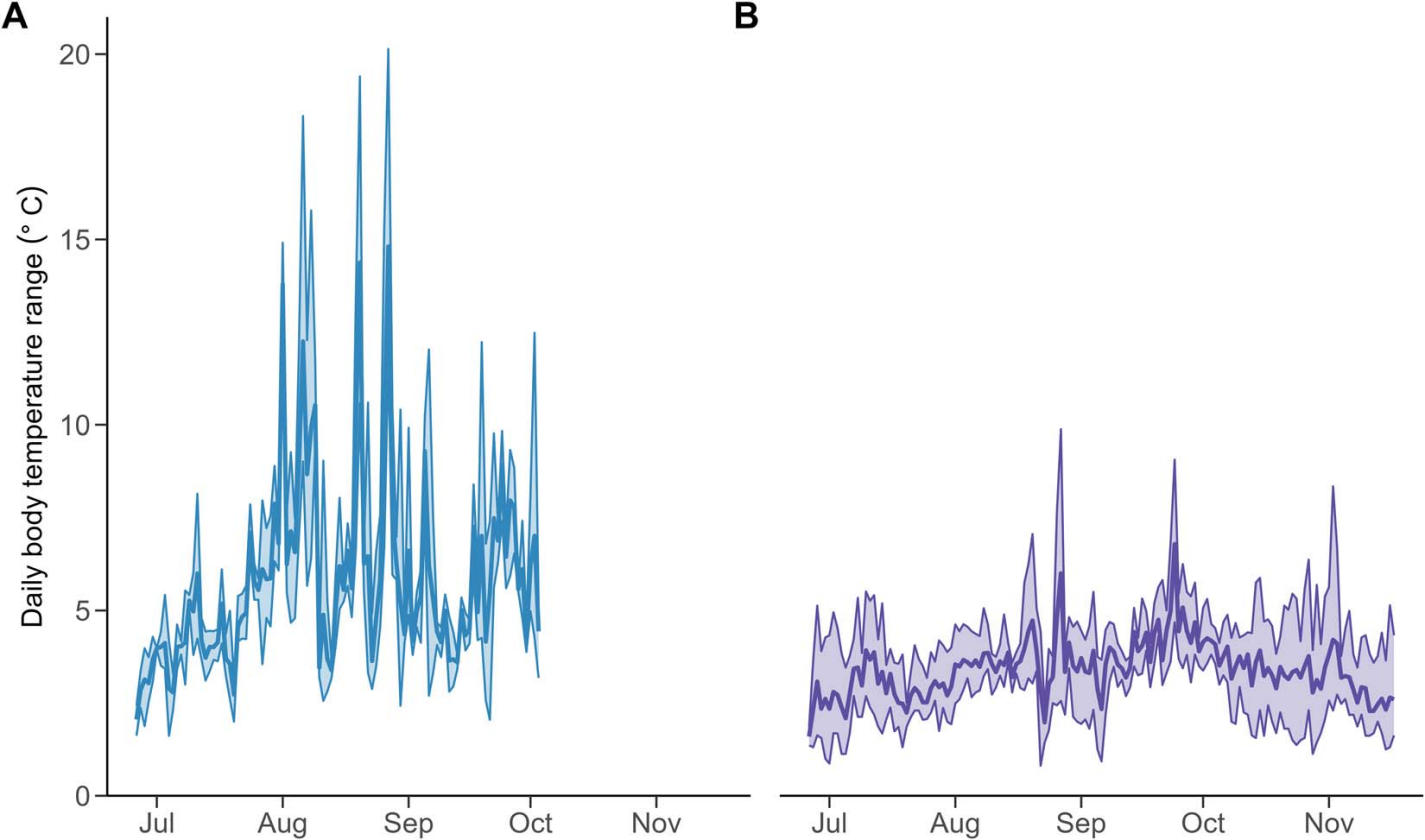
(**A**) Mean T_b_ variation of male gliders (blue solid line) increased between August and October. (**B**) Mean T_b_ variation of females (purple solid line) showed only a couple of peaks during the same time frame and was largely similar across the entire study. Shaded area shows the overall range (min and max) of daily T_b_ variation across all individuals.

## Discussion

Our study covered two energetically demanding time periods for Krefft’s gliders: winter when females were gestating and T_a_s were low and thermoregulatory costs were high, and spring when females were lactating. Via the use of HR and T_b_ biologging, we were able to investigate the costs of different reproductive stages in gliders at a very fine timescale. Surprisingly, under semi-natural conditions gestation was the costliest period for gliders, likely due to the increased thermoregulatory costs needed to maintain a high and stable T_b_ to support the development of young during winter. We found that by timing lactation during warmer months and increasing variation in daily T_b_, females were able to reduce the costs of lactation substantially, contradicting our expectations that lactation is the most energetically demanding reproductive stage.

Overall, captive sugar gliders in our study used substantially less energy than previously reported for free-ranging sugar gliders. The average estimated DEE across all individuals of 88.7 kJ per day was approximately half that found in previous studies that employed DLW (169 kJ per day (Nagy & Suckling, 1985); 158 kJ per day (Quin *et al.,* 2010)). The differences in our findings most likely reflect the overall lower activity levels in our captive gliders. Free-ranging gliders are known to be active across the entire night (Körtner & Geiser, 2000) and have an average home range of 4.7 ha (up to 14 ha; Jackson, 2015). Given that our gliders had nightly access to food and water, their energy expenditure likely consisted primarily of thermoregulatory demands and any additional reproductive costs. Indeed, we found that over the course of our study, estimated DEE declined as seasons transitioned from winter to spring associated with increasing daily T_a_, and that females capitalized on this by generally timing lactation during periods where costs of thermoregulation were reduced.

Due to the immense costs of milk production, lactation is typically the most energetically expensive period in the reproductive cycle of mammals, especially in marsupials. In homeothermic free-ranging ringtail possums (*Pseudocheirus peregrinus*) for example, DEE during late stage lactation increased by ~35% compared to during the non-reproductive stage (Munks & Green, 1995). To balance this cost, most mammals time lactation to coincide with periods of high food abundance. In heterothermic species, flexibility in thermoregulation can prolong reproduction to align reproductive phases more precisely with beneficial environmental conditions (McAllan & Geiser, 2014), like periods of low thermoregulatory cost, as we found for the gliders in our study. However, the extent to which T_b_ fluctuates and the expression of torpor during different reproductive phases varies with different species (Csada & Brigham, 1994; Willis *et al.,* 2006; Stawski, 2010; McAllan & Geiser, 2014). A tightening of T_b_ control during gestation and an increase in T_b_ variation during lactation, as we found for our gliders, is common in marsupial petaurid heterotherms (McAllan & Geiser, 2014). For example, Krefft’s gliders in the wild defended a high and stable T_b_ during gestation and only employed torpor during lactation (Geiser *et al.,* 2008). Conversely, captive yellow-footed antechinus (*Antechinus flavipes*), a marsupial dasyurid, restricted daily T_b_ changes during parturition and early stage lactation but regularly entered torpor when gestating (Stawski & Rojas, 2016). This suggests that marsupial petaurids and dasyurids differ in their energetic strategies during reproduction. The defence of a high and stable T_b_ during periods of low T_a_ most likely explain why gestating females in our study had the highest estimated DEE overall.

Increased T_b_ variability of even just a few degrees typically results in energetic savings, and this was evidenced by both females and males in our study. A daily T_b_ variation of up to 10°C resulted in a 15.3 kJ savings in mean estimated DEE for lactating females compared to gestating females, and near 20 kJ per day for males. It is clear that energy savings from flexible thermoregulation are maximized during periods of low T_a_ where thermoregulatory costs are highest. Gliders used torpor most often and on days when minimum temperatures were lowest (<15°C) and saved up to 84% during torpor when T_a_ fell below zero. Yet our results show that even small variations in T_b_ during mild conditions can be sufficient to balance, and even reduce, the mostly costly of life history stages. This is somewhat surprising considering that other heterothermic marsupials known to use torpor during gestation but not during lactation, such as the brown antechinus *(Antechinus stuartii),* still show an increase in energy expenditure of 30% during late lactation compared to post-lactation in captive settings (Westman *et al.,* 2002). If we assumed a similar proportional cost of lactation for our gliders, mean DEE in gliders from our study should have peaked near 135 kJ per day during late lactation instead of ~97 kJ per day. However, many dasyurid species such as antechinus have very large litters (6–10), whereas gliders have smaller litters (1–2). It should be noted that each of our enclosures housed four gliders, unlike other studies where pregnant females are housed alone, so we cannot be certain of the specific food intake for the females. It is plausible that the substantial reduction in estimated DEE we report was also influenced by mass loss in the females, as has been reported for free ranging gliders during lactation (Suckling, 1984). Alternatively, lactating females in our study may have eaten proportionately more food than the other gliders and our results reflect a further reduction in activity during lactation. In either case, the plasticity in thermoregulation and reproductive timing in these gliders enabled the successful rearing of 70% of the young to the nestling stage, similar to reports for wild gliders (59–84%; Suckling, 1984). Krefft’s gliders can be diestrous depending on the timing of their first pregnancy and environmental conditions. Given our findings, it seems likely that their broader physiological flexibility has enabled them to survive inclement conditions and may provide a buffer to population persistence under future climate scenarios.

Our findings also differ in another aspect from field studies conducted on Krefft’s gliders that found no differences in energy expenditure between the sexes, even with females carrying pouch young (Nagy & Suckling, 1985; Quin *et al.,* 2010). Surprisingly, Quin *et al.* (2010) also found no seasonal differences in FMR across spring, summer and autumn. The overriding costs of activity and foraging, and the limitations of data collection associated with DLW in these studies (MR integrated over only 1–2 days) may explain the inability to detect differences between sexes and across seasons. It is also plausible that free-ranging Krefft’s gliders may compensate for seasonal changes in T_a_ by altering their behaviour (such as huddling) or through the use of torpor, leading to consistent energy expenditure across the year (Quin *et al.,* 2010; Nowack & Geiser, 2016). For example, squirrel gliders (*Petaurus norfolcensis*) were found to use torpor opportunistically rather than regularly throughout the year, and increased torpor use in spring likely due to reproductive state rather than in response to environmental variables (Dausmann *et al.,* 2023). The observed seasonal changes in estimated DEE in our study and the associated thermoregulatory costs with changes in T_a_ may have overridden the energetic costs associated with reproductive state, as no significant differences were found with the exception of gestating females. This supports the hypothesis that within individual variation over time drives patterns of energy management, something that was potentially unable to be observed in previous studies due to the integrative nature of the DLW method (Halsey *et al.,* 2019). Furthermore, this highlights the benefits of physiological biologging where fine timescale measurements enable more comprehensive assessment of physiological and life history states (Currie *et al.,* 2026).

## Conclusion

By confirming the relationship between HR and oxygen consumption under laboratory conditions, we were able to estimate DEE of captive Krefft’s gliders in outdoor enclosures exposed to natural weather variation during their reproductive period. Our findings suggest that female gliders trade off thermoregulatory and reproductive requirements differently depending on the stage of reproduction. Tight thermoregulatory control coupled with low T_a_ appears to drive our findings of the high energetic costs of gestation. However, females appear able to compensate for the increased costs of milk production during late lactation by reduced thermoregulatory demands in spring alongside greater flexibility in daily T_b_. These findings were only possible due to the integration of both HR and T_b_ measurements and emphasize the importance of using multiple sensors in biologging studies (Currie *et al.,* 2026). We showed that the interaction between T_b_ and HR was important in improving predictions of oxygen consumption from HR in a heterothermic species. Our established model can now be used to predict energy expenditure from HR and Tb in future ecophysiology studies on free-ranging gliders. Understanding the energetic landscape of species under different life history states is vital for comprehensive conservation management. More specifically, these insights can be used to improve dynamic energy budget models, which rely on accurate parameterization of costs of reproduction and associated offspring survival alongside mechanisms used by individuals to balance their energy budgets. These models can then be used to estimate species persistence under scenarios relevant to their conservation (e.g. climate or land-use change) to best optimize management efforts.

## Supplementary Material

Web_Material_coag072

## Data Availability

Data from this study are accessible from the Supplementary material.
