# Supplementary material for: Heart rate biologging as a tool to understand the energetic needs of life history states
Source: Conserv Physiol. 2026 Sep 28;14(1):coag072. doi: 10.1093/conphys/coag072 (PMC13618255; doi:10.1093/conphys/coag072)
Supplement: Supplementary file 1 [file web_material_coag072.docx]

**Supplementary material for:**

**Heart rate biologging as a tool to understand the energetic needs of life history states**

Clare Stawski, Fritz Geiser, Gerhard Körtner, Shannon Currie

**Table S1.** Summary statistics of the absolute difference between logger determined heart rate and heart rate measured via ECG verification. Data is grouped via StarOddi’s automated QI classification. There was no clear pattern indicating that QI 0 & 1 were more accurate HR values than QI 2 & 3.

| **Glider** | **QI** | **Logger HR versus ECG verified HR (bpm)** | | | |
| --- | --- | --- | --- | --- | --- |
|  |  | **Minimum** | **Maximum** | **Mean** | **Median** |
| F1 | 0 | 1 | 273 | 26 | 4 |
| F2 | 0 | - | - | - | - |
| F3 | 0 | 0 | 183 | 12 | 2 |
| F4 | 0 | - | - | - | - |
| M1 | 0 | 3 | 228 | 24 | 9 |
| M2 | 0 | 1 | 240 | 63 | 14 |
| M3 | 0 | 0 | 327 | 33 | 2 |
| F1 | 1 | 1 | 358 | 98 | 24 |
| F2 | 1 | 1 | 66 | 24 | 14 |
| F3 | 1 | 1 | 178 | 29 | 17 |
| F4 | 1 | 9 | 62 | 34 | 29 |
| M1 | 1 | 1 | 179 | 45 | 35 |
| M2 | 1 | 3 | 402 | 62 | 34 |
| M3 | 1 | 0 | 210 | 44 | 25 |
| F1 | 2 | 1 | 334 | 117 | 65 |
| F2 | 2 | 2 | 264 | 67 | 24 |
| F3 | 2 | 3 | 355 | 65 | 31 |
| F4 | 2 | 7 | 57 | 31 | 30 |
| M1 | 2 | 4 | 287 | 59 | 39 |
| M2 | 2 | 2 | 432 | 68 | 46 |
| M3 | 2 | 0 | 259 | 68 | 41 |
| F1 | 3 | 0 | 376 | 45 | 11 |
| F2 | 3 | 1 | 58 | 19 | 15 |
| F3 | 3 | 0 | 217 | 24 | 8 |
| F4 | 3 | 0 | 442 | 41 | 15 |
| M1 | 3 | 0 | 360 | 71 | 37 |
| M2 | 3 | 4 | 257 | 94 | 78 |
| M3 | 3 | 0 | 184 | 48 | 13 |

**Figure S1. A)** Boxplots showing the distribution of heart rate values as a function of quality assurance classifications (QI0 - 3) for HR measurements following max-min filtering (N = 30571; see Methods for filtering details). All QI categories had representative HR measurements from across the variation in HR, although HR data classified as QI3 was higher on average than the other classifications (tukey’s-adjusted post-hoc, all p-values <0.05). **B)** Proportionate distributions of QI classifications compared across reproductive states for all data. As males were the only individuals for which intraperitoneal implantation occurred, only data from subcutaneously implanted loggers were compared for consistency.

**Figure S2.** **A)** Plotted relationship between logger determined heart rate and heart rate measured via ECG verification (N = 600). Colours indicate logger placement and dashed line indicates line of equality. There was no clear difference between the ability to detect HR based on logger placement. **B)** Comparison of the proportions of quality assurance classifications (QI0 - 3) of all HR data based on logger placement (N = 38011). Colours indicate QI categories. There were proportionately fewer data classified as QI3 when loggers were implanted intraperitoneally.

**Figure S3**. Marginal effects plot showing the predicted relationship between heart rate and oxygen consumption across representative body temperatures. Colours and line types indicate the different body temperatures, shading indicated the 95% confidence intervals around our predictions derived from bootstrapping). The selected body temperatures represent the 10^th^, 50^th^ and 90^th^ percentile of the data used to generate the model. As body temperature increases the slope of the relationship between heart rate and oxygen consumption increases.

**Figure S4.** Example model predictions from the model including HR data from all quality assurance categories (solid black line) compared with a model excluding HR data from QI3. The substantial overlap between the two models suggests that exclusion of HR data classified as QI3 does not improve model performance.

**Table S2.** Mean daily energy expenditure of gliders during different reproductive states.

| **Reproductive state** | **Mean daily energy expenditure (kJ d^-1^) ± S.D.** | **Mean daily range in heart rate (bpm) ± S.D.** |
| --- | --- | --- |
| Male | 81.8 ± 15.5 | 423 ± 88 |
| Non reproductive female | 99.5 ± 8.9 | 391 ± 64 |
| Gestating | 108.0 ± 10.0 | 395 ± 88 |
| Lactating (teat) | 97.4 ± 7.9 | 400 ± 73 |
| Lactating | 87.1 ± 11.3 | 432 ± 100 |
